# A Time-Domain Signal Processing Algorithm for Data-Driven Drive-by Inspection Methods: An Experimental Study

**DOI:** 10.3390/ma16072624

**Published:** 2023-03-26

**Authors:** Yifu Lan, Zhenkun Li, Weiwei Lin

**Affiliations:** Department of Civil Engineering, Aalto University, 02150 Espoo, Finland

**Keywords:** signal processing, structural health monitoring, drive-by bridge inspection, vehicle-bridge interaction, machine learning, sliding window

## Abstract

Constructional material deterioration and member damage can cause changes in the dynamic characteristics of bridge structures, and such changes can be tracked in the responses of passing vehicles via the vehicle-bridge interaction (VBI). Though data-driven methods have shown promising results in damage inspection for drive-by methods, there is still much room for improvement in their performance. Given this background, this paper proposes a novel time-domain signal processing algorithm for the raw vehicle acceleration data of data-driven drive-by inspection methods. To achieve the best data processing performance, an optimizing strategy is designed to automatically search for the optimal parameters, tuning the algorithm. The proposed method intentionally overcomes the difficulties in the application of drive-by methods, such as measurement noise, speed variance, and enormous data volumes. Meanwhile, the use of this method can greatly improve the accuracy and efficiency of Machine Learning (ML) models in vehicle-based damage detection. It consists of a filtering process to denoise the data, a pooling process to reduce data redundancy, and an optimizing procedure to maximize algorithm performance. A dataset is obtained to validate the proposed algorithm through laboratory experiments with a scale truck model and a steel beam. The results show that, compared to using raw data, the present algorithm can increase the average accuracy by 12.2–15.0%, and the average efficiency by 35.7–96.7% for different damaged cases and ML models. Additionally, the functions of filtering and pooling operations, the influence of window function parameters, as well as the performance of different sensor locations, are also investigated in the paper. The goal is to present a signal processing algorithm for data-driven drive-by inspection methods to improve their detection performance of bridge damage caused by material deterioration or structural change.

## 1. Introduction

In the U.S., 42% of all bridges are at least 50 years old, and structural deficiencies (e.g., material deterioration) have been recorded in 46,154, or 7.5% of the nation’s bridges [1]. In Europe, it has been reported that most bridges were constructed between 1945 and 1965, and some of them have recently experienced aging and deterioration problems [2]. As a tragic example, the Genoa bridge collapse in Italy claimed the lives of 43 people in 2018, mainly due to poor maintenance with difficulties in inspection [3,4]. Bridge structural failure has emerged as a worldwide issue, and it is essential to build effective bridge health monitoring systems that can detect damage in its early phases [5].

Damage is generally considered to be a change in effective material properties due to cracks, spalling, corrosion, delamination, voids, etc. Traditional inspection methods often require on-site inspections, such as visual monitoring by experienced experts [6]. However, in addition to the substantial labor costs and operational interruptions, these methods have drawbacks that they may fail to identify internal damage of the construction materials. On the other hand, the internal damage will induce changes in the vibrational properties, and vibration-based detection techniques can thus address these shortcomings [7]. Nevertheless, due to the high costs of installing and maintaining sensors, direct methods of vibration-based structural health monitoring (SHM) have long been regarded as expensive technologies [8]. Furthermore, because the equipment is permanently attached to the bridge as a customized SHM system, it could be challenging to transfer one monitoring framework to other bridges [9]. Given these, it is necessary to develop an alternative technique without instrumenting the bridge.

Due to its advantages in mobility, economics, and efficiency, the drive-by bridge inspection method, an indirect SHM approach, has recently attracted a lot of interest [10]. It was initially proposed by Yang et al. [11], who analytically retrieved the fundamental frequency of the bridge from the vibrational responses of a passing vehicle. It simply requires a few sensors mounted on the vehicle rather than instrumentation on the bridge, providing great practicability [12]. Through numerical simulations, laboratory experiments, and field tests, studies have validated the drive-by method’s potential for obtaining bridge modal parameters over the past two decades, such as fundamental frequencies and mode shapes [13,14,15,16,17,18,19,20]. Changes in modal parameters can be an indication of damage (e.g., material deterioration or structural change), which is known as the modal parameter-based method [21].

However, the drive-by methods based on modal parameters generally struggle to achieve satisfactory performance in damage detection due to several reasons. First, the natural frequencies used as damage indicators in several studies are easily influenced by external factors such as temperatures, which may conceal the change induced by damage [22]. Second, the other frequently used indicators, such as mode shapes or their derivations, are often vulnerable to measurement errors/noise, masking changes from small-scale damage [23]. Third, there is a potential risk of human bias because such approaches heavily rely on the researchers’ expertise and experience [24]. Furthermore, the drive-by methods based on modal parameters might have difficulties quantifying the severity of damage [25]. Although there are methods based on moving force and displacement profiles, etc. [26,27,28,29], similar problems have been noticed, and they usually lack experimental validation.

As an active area of research, data-driven approaches have recently gained popularity. They have demonstrated great achievements in indirect SHM frameworks. For example, Cerda et al. [30] successfully identified various bridge damage cases using Support Vector Machines (SVM) by inputting vehicle frequency domain data. Liu et al. [31] achieved damage quantification of bridges by utilizing stacked autoencoders as dimensionality reduction techniques to explore the vehicle’s full bandwidth frequency response. Sarwar and Cantero [32] effectively detected bridge damage by using a fleet of vehicles’ responses and adopting deep autoencoders as feature extraction techniques. These methods, however, rely on ML techniques used as signal processing tools for feature extraction. Since the extracted features from ML techniques are not often transferable to other bridges [33], one concern is that such approaches might bind the monitoring framework to a specific structure or system. Furthermore, most of the current methods are based on the frequency domain. The transformation of signals from the time domain to the frequency domain can result in information losses [34]. The information losses are primarily the non-stationary features of signals that vary with time, whereas vibrational signals in engineering practice are usually non-stationary. Time-domain signals can effectively display the temporal characteristics of vibration as they change over time. They may also be related to the damage, particularly in the case of minor or local damage.

Some researchers have suggested that the time-domain signal contains richer damage information and is thus more sensitive to structural damage [23,24]. As an example of machine learning-based indirect SHM using time-domain data, Lan et al. [35] accurately detected a 1% structural mass increase in bridges via an optimized AdaBoost-linear SVM, where only raw acceleration data from a vehicle were utilized as direct input in their study. The authors believe that preserving time-domain features when processing acceleration data can be beneficial to damage detection. However, most of the data processing methods currently applied to the drive-by method do not pay sufficient attention to the temporal information. In general, there are several challenges in the processing of time-domain signals from drive-by measurements. First, a drive-by measurement inevitably contains noise (e.g., environmental noise) in the time domain, which could affect the results. Second, it is often difficult to ensure a constant vehicle speed, resulting in irregular data sizes, while the same data size is important for many ML methods. Third, the time-domain signals usually have massive amounts of data, requiring large computational resources. Addressing these issues is key to processing the time-domain responses from a drive-by measurement.

This paper proposes a novel time-domain signal processing algorithm for the raw vehicle acceleration data of data-driven drive-by inspection methods. It aims to provide an efficient, transferable, and easy-to-use signal processing method for the indirect SHM framework, which can significantly improve the accuracy and efficiency of ML models in damage detection. The data processing method consists of procedures for filtering and pooling; they are sliding window-based methods, in which the former aims to denoise data, while the latter seeks to equalize the data size and reduce data redundancy. In addition, an optimizing strategy is designed to automatically search for the optimal parameters to tune the algorithm, maximizing the performance in data processing. A dataset is obtained to validate the proposed algorithm through laboratory experiments with a scale truck model and a steel beam. The performance of the methodology is illustrated by its accuracy and efficiency improvement in damage detection with ML models such as SVM. The functions of filtering and pooling processes, the influence of window function parameters, as well as the performance of different sensor locations are also investigated in the study. The final goal is to present a signal processing algorithm for data-driven drive-by inspection methods to improve their detection performance of bridge damage caused by material deterioration or structural change.

## 2. Materials and Methods

### 2.1. Data-Driven SHM Framework

**Figure 1:**
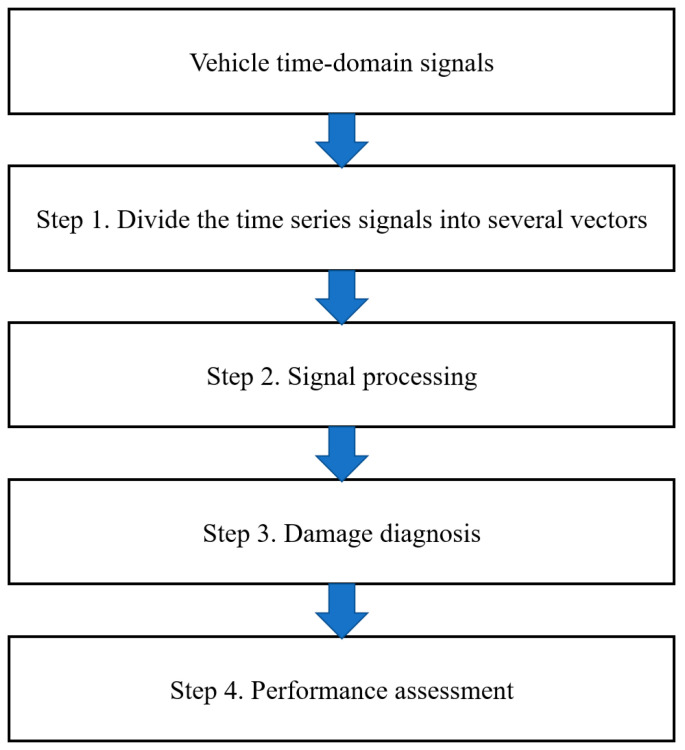
Designed SHM framework.

A data-driven drive-by SHM framework can be considered as a four-step process, as presented in Figure 1. In step 1, the raw acceleration signals collected from a passing vehicle are divided into vectors corresponding to the number of times the vehicle runs on the bridge. In step 2, the time-domain signals, during which the whole car is on the bridge, are processed to remove noise, redundant information, etc. In step 3, the processed vectors are then used as inputs for ML models for damage diagnosis; they are usually split into training and test sets, and labels are required in supervised/semi-supervised learning models. In step 4, the performance of ML models is evaluated based on their accuracy, efficiency, etc. The proposed data processing methods are implemented in step 2, which aim to improve the accuracy and efficiency of the data-driven drive-by SHM framework in general.

### 2.2. Filtering Procedure

**Figure 2:**
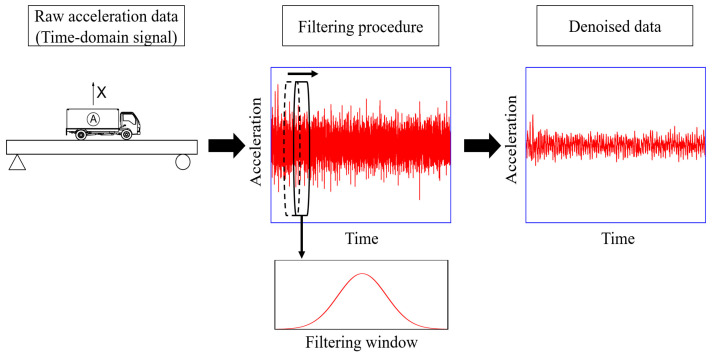
Data acquisition with filtering operation.

The signal processing methods involve a filtering procedure and a pooling procedure. As a sliding window technique [36], the data acquisition with filtering operation can be referred to in Figure 2. In the process, each signal in the input data is scanned with a template (or kernel, mask); the signal value is replaced with the neighborhood’s weighted average signal value, which is determined by the window function (see Equation (1)). In the equation, i represents the i-th signal in the time-domain input, I(i); m denotes the length of the window function, W(n); the window function is suggested to be selected according to the dominant noise. The Gaussian function is chosen as the window function (see Equation (2)) in this study, as white noise is the main source of noise in a normal laboratory environment. In the equation, the expectation, µ, is zero and the deviation, σ, is determined by the optimizing procedure. Based on some instances in the literature [37,38], the environmental noise can be theoretically added to the acceleration signal in the form of Equation (3). In the equation, ${\ddot{y}}_{p}$ is the polluted acceleration data; $E_{p}$ represents the noise level; $N_{s}$ denotes the standard normal distribution, and $\sigma_{{\ddot{y}}_{v}}$ is the standard deviation of the vehicle response ${\ddot{y}}_{v}$. In this case, the Gaussian kernel filtering operation could perform well. The influence of different deviation values will be discussed below. It should be noted that the operation will begin with m/2 zeros padding the left end of the input data to align it with the window function. The filtering procedure aims to obtain denoised data by denoising the raw time-domain signals; it does not change the data size.

(1) $$f(i)=\sum_{n=1}^{m}I(i+n)W(n)$$

(2) $$W\left(x\right)=\frac{1}{\sqrt{2\pi }\sigma }exp\left(-\frac{(x-µ{)}^{2}}{2\sigma^{2}}\right)$$

(3) $${\ddot{y}}_{p}={\ddot{y}}_{v}+E_{p}N_{s}\sigma_{{\ddot{y}}_{v}}$$

### 2.3. Pooling Procedure

**Figure 3:**
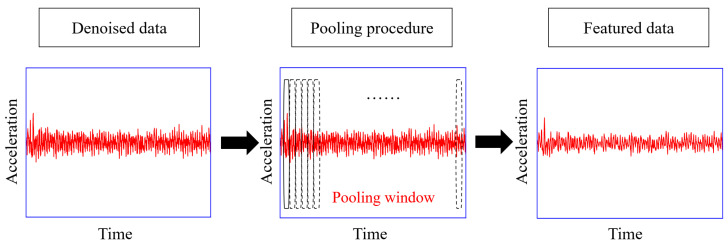
Pooling procedure.

**Figure 4:**
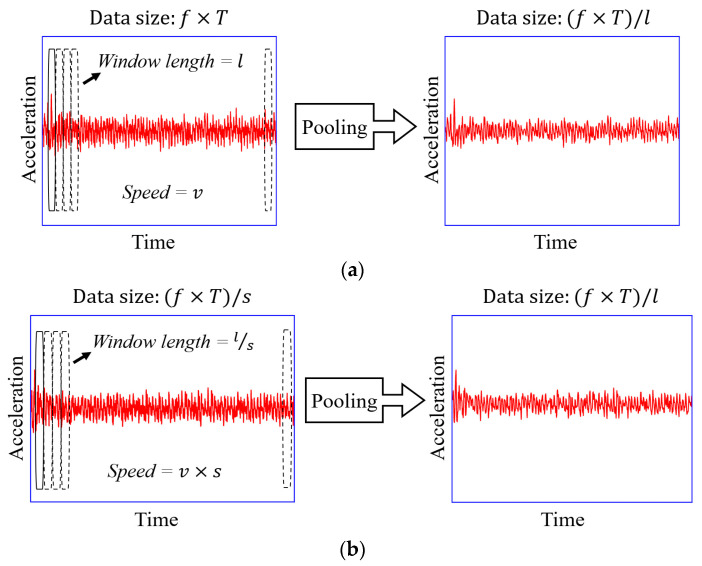
Pooling operation for vehicle’s time-domain signals with different speeds. (**a**) Speed = v, (**b**) Speed = s×v.

The pooling operation further processes the denoised data from the filtering procedure, as shown in Figure 3. The concept of the pooling procedure can refer to the pooling layer in the neural network [39], where the pooling operation retrieves the representative features, such as the maximum value and mean value, within a certain neighborhood along the time-series direction. The data’s characteristics remain mostly unchanged throughout the pooling process, despite a reduction in data size [40]. Figure 4a illustrates how the operation pools the data of the vehicle traveling at speed v. The passing time is T, the sampling frequency is f, and the data size is f×T; Figure 4b shows the pooling process on the car with speed s×v, in which the speed ratio of the run to the first is s, and its data size is computed as (f×T)/s. Through the pooling process, data of the same size can be acquired by modifying the window length, l, according to speed variations; the data size after pooling, $\overset{-}{N}$, becomes (f×T)/l. $\overset{-}{N}$ is automatically chosen by the optimizing procedure in this study, where l is assigned based on the vehicular speed in each run (stride = window length). Zeroes will be automatically appended to the end of any data that cannot be split by $\overset{-}{N}$; the edge data has little effect on the results. Max pooling is adopted in this study since it has been proven to be more informative in practice [39]. When processing acceleration signals, the max pooling can preserve temporal information, such as local amplitude peaks, to a great extent. The effects of $\overset{-}{N}$ or l will be also discussed below. It should be noted that the speed difference in such a method should not be too large; some earlier research [41,42,43] found an empirically acceptable velocity difference of roughly 40%. The pooling process can further mitigate noise, equalize data size, and reduce computational costs.

### 2.4. Dataset Format and ML Models

**Figure 5:**
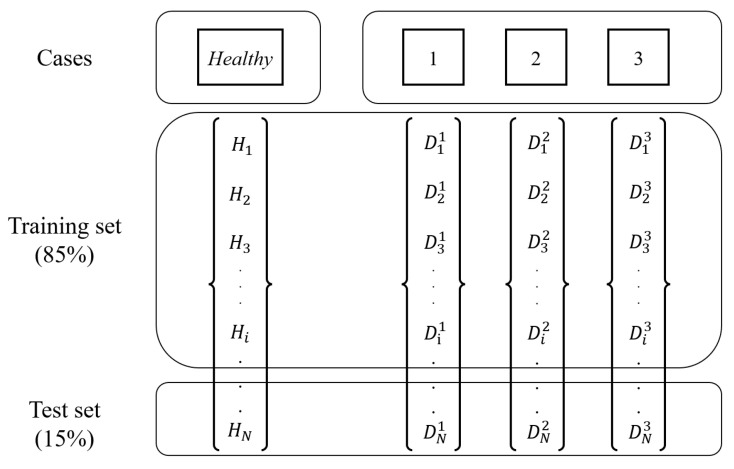
Format of the dataset.

In the data-driven SHM framework, ML models learn to identify damage by feeding samples from the training set, and their performance can be evaluated by their accuracy in the test set. The accuracy, AC, can be calculated using Equation (4). In the equation, TP, TN, FP, and FN represent true positive, true negative, false positive, and false negative predictions in test samples, respectively. As presented in Figure 5, the dataset from different cases is split into training and test sets at a ratio of 85% to 15% in this study, and the performance of each ML method is assessed using five-fold cross-validation. In the figure, N denotes the total vehicle runs for each case, and the superscript numbers (e.g., 1, 2, 3) correspond to different damage cases.

(4) $$AC=\frac{TP+TN}{TP+TN+FP+FN}$$

In this study, five different ML models are chosen, which are linear-SVM, RBF-SVM, Gaussian Process (GP), Artificial Neural Network (ANN), and Random Forest (RF). They are ML models often employed in SHM problems. The performance of the proposed signal processing algorithm is assessed by its improvement in accuracy and efficiency of damage detection with different ML methods. The descriptions of the ML models used in this paper are as follows:

Linear-SVM: One of the most robust and accurate models of well-known ML algorithms is SVM [44]. The goal of linear-SVM is to find separating hyperplanes that can separate the dataset as reliably as possible into the distinct data classes. Ideally, when the data are completely linearly separable, the hyperplanes will be as far as possible from the nearest elements of the classes.

RBF-SVM: RBF-SVM, as one of the nonlinear SVMs, replaces hyperplanes with Gaussian manifolds, but the basic principle remains the same [44]. One can adapt SVM to be a nonlinear classifier, which allows SVM to separate nonlinearly separable support vectors. The codes and implementation of SVMs can refer to LIBSVM: A library for support vector machines [45].

GP: GP is an infinite-dimensional generalization of multivariate normal distributions and can be used for classification and regression. It is a type of kernel model, similar to SVM, but unlike SVM, it can predict highly calibrated class membership probabilities, although the choice and configuration of the kernel used at the heart of the method can be challenging. Its codes and implementation can refer to Gaussian Processes for Machine Learning [46].

ANN: ANN is based on a collection of connected units or nodes called artificial neurons, which loosely resemble the neurons in a biological brain. Each connection, like the synapses in a biological brain, can transmit a signal to other neurons. Typically, neurons are aggregated into layers. Different layers may perform different transformations on their inputs. Signals travel from the first layer (the input layer) to the last layer (the output layer), possibly after traversing the layers multiple times. Its codes and implementation can refer to Deep Learning [40].

RF: RF is an extension of the bagging method, which uses feature randomness in addition to bagging to produce an uncorrelated forest of decision trees [47]. For classification problems, the output of RF is the class selected by most trees. RF generally outperforms decision trees, but its performance can be affected by data characteristics. Its codes and implementation can refer to Random Forests [48].

### 2.5. Optimizing Procedure

**Figure 6:**
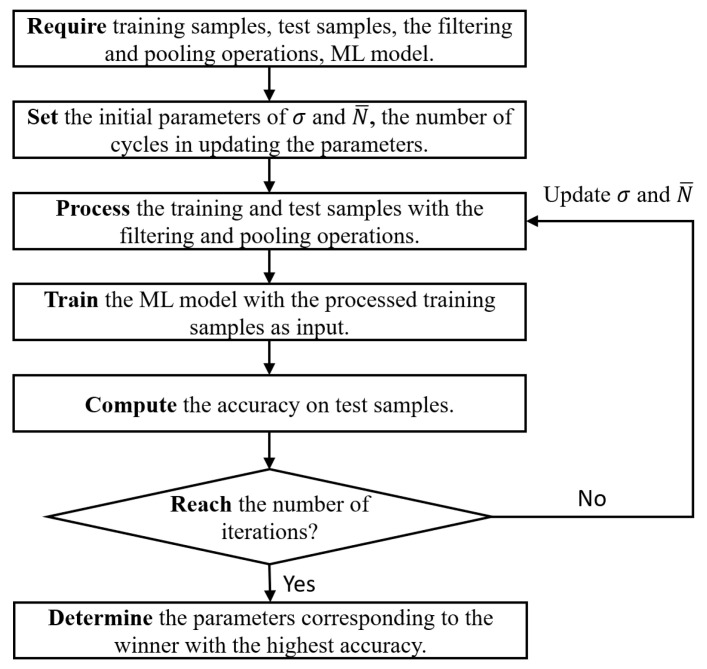
Optimizing process.

As can be seen from the above process, the performance of the filtering and pooling operations is affected by their parameters, σ and $\overset{-}{N}$ (or l). There may also be optimum σ and $\overset{-}{N}$ values that can lead to the best performance in data processing. Given the ML model, training and testing samples, the optimizing procedure can search for these optimum parameters. Figure 6 shows the complete optimizing process, which can be regarded as a loop program. The initial parameters of σ and $\overset{-}{N}$, and the number of cycles in updating them are set to 0.2, 4000, and 200, respectively, in this study. In each loop, the ML model is trained using the processed training samples as input, after which the accuracy on test samples is computed. The one with the highest accuracy is chosen as the winner, and then the optimum parameters for data processing can be accordingly determined. The loop is stopped when the number of iterations is reached. This way, the optimizing procedure can achieve automatic parameter tuning of the data processing. The optimal tuning algorithm, as an auxiliary tool, can be understood to a certain extent as a “grid search” strategy [49] customized for the proposed filtering and pooling operations. The selection of parameters in this study can be seen in the “Results and Analysis” section, which provides the greatest score among all the candidate parameters, but, for other structures, they may be different. Based on these results, 200 iterations are sufficient for this experimental database or a small database, while it is usually necessary to choose a greater number of iterations for larger databases. It is worth noting that this optimizing program requires labelled samples. For data with unlabeled samples, the above signal processing methods (i.e., the filtering and pooling operations) are still applicable, but this optimization method cannot be directly used.

## 3. Experimental Program

Laboratory experiments were performed to validate the proposed algorithm using a HEA400 steel beam and a scale truck model with an engine. Acceleration data obtained from the vehicle sensor were utilized to construct the dataset. In this study, damage cases were simulated by adding weights to the bridge model, known as “artificial damage”, to avoid permanent or irreversible destruction to the material, which has been proven feasible by many studies [31,35,43,50]. The data acquisition system in this study was driven by a PC, connected to the sensor by wires, and had a sampling rate of 2 kHz; the sensor was manufactured by Bruel and Kjaer (TYPE 4371) [51].

### 3.1. Vehicle Model

**Figure 7:**
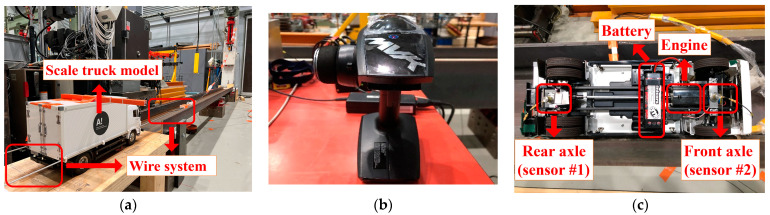
Vehicle model setup. (**a**) Scale truck model, (**b**) Remote controller, (**c**) Sensor installation.

**Figure 8:**
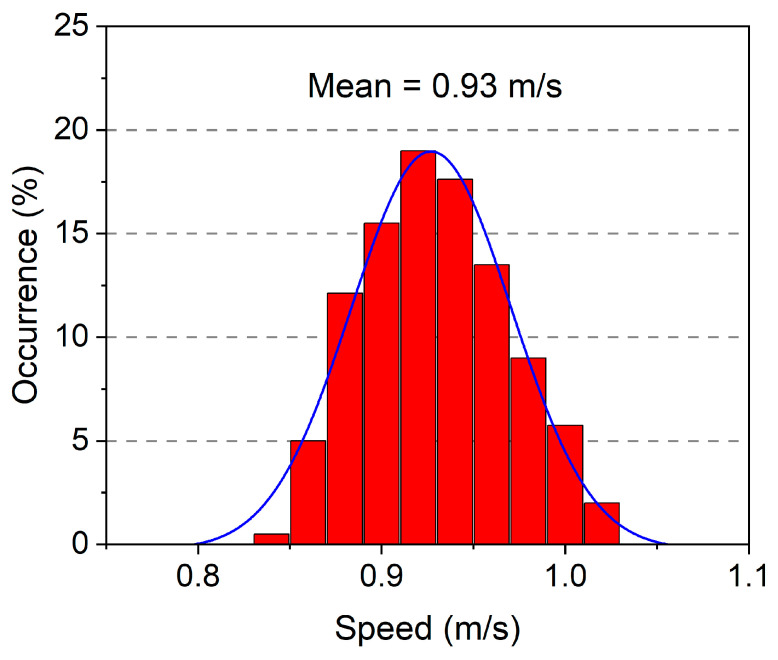
Statistics distribution for velocities.

A Tamiya Mercedes-Benz 1850L is employed as the vehicle model in the study, as shown in Figure 7a. Tamiya is a Japanese manufacturer of car models, known for its precise scale details and excellent quality. This is a 1:14 scale model of a full-sized truck, and its weight is experimentally measured as 4.05 kg. In addition, the vehicle body is loaded with a 5 kg weight, so the weight of the whole vehicle is 9.05 kg. A wire system is used to guide the vehicle to travel through the beam in a straight line and along the same path. The car model uses a battery-driven motor and is operated with a remote controller (see Figure 7b) to move at a relatively constant speed. Two accelerometers are mounted on the rear axle (sensor#1) and the front axle (sensor#2), respectively, as indicated in Figure 7c. In the ML-based drive-by inspection methods, the vehicle configuration should be consistent in multiple measurements, and its speed difference should not be too large (generally less than 40%) for different tests [35,41,42]. A relatively low speed is recommended according to some previous studies [18]. As may be seen in Figure 8, the experiment’s statistical distribution of velocities nearly conforms to the normal distribution. The mean speed is 0.93 m/s, and the velocity distribution ranges from 0.84 m/s to 1.02 m/s. The maximum to minimum velocity ratio is 121.4%, which is less than the acceptable speed difference of 40%.

### 3.2. Bridge Model

**Figure 9:**
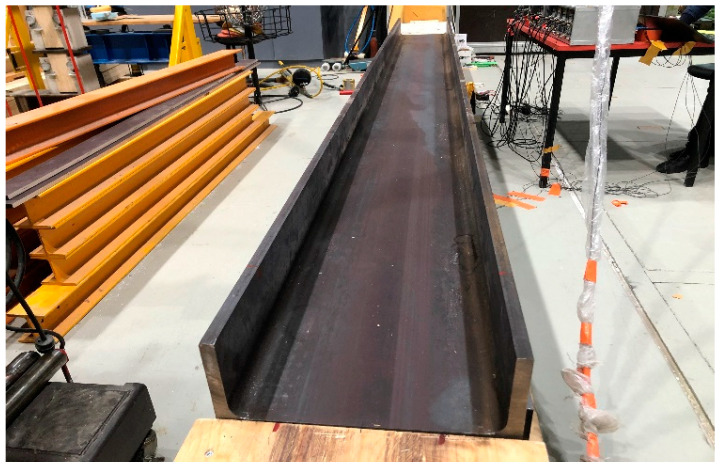
Simply supported bridge model.

**Figure 10:**
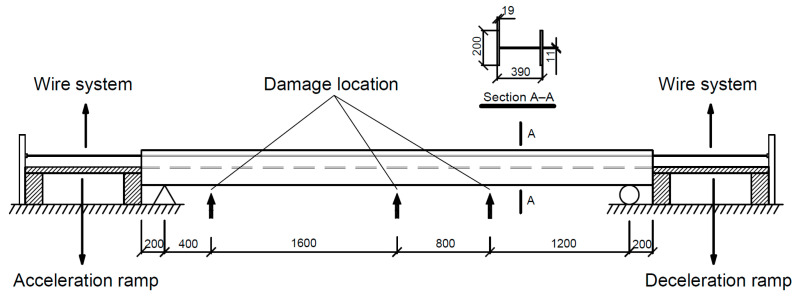
Details of the bridge model setup (unit: mm).

**Figure 11:**
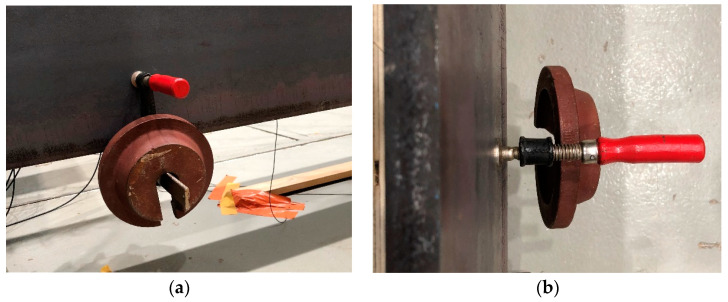
Mass placement. (**a**) front view, (**b**) side view.

A HEA400 steel beam is utilized as the simply supported bridge model in the experiment, as shown in Figure 9. The details of the bridge model, including the sectional dimensions, are shown in Figure 10. In addition to the wire system, the experimental setup also includes acceleration and deceleration ramps, ensuring that a stable speed can be approached before reaching the beam. The physical properties of the beam are provided as follows: elastic modulus E = 199 GPa; density ρ = 7.85 × 10^3^ kg/m^3^; total length L = 4.4 m (span length $L_{s}$= 4 m); section area A = 15898 mm^2^; and moment of inertia I = 8.564 × 10^7^ mm^4^. Figure 11 shows the additional masses used as artificial damage. The mass placed in different positions on the beam represents different damage locations; the case details will be discussed later.

### 3.3. Damage Cases

In the ML-based drive-by inspection framework, the vehicle is usually required to be repeatedly driven across the bridge to obtain the dataset. Table 1 shows all state scenarios employed in the experiment, where the healthy case is “case 0”. The damaged cases are collected from three damage locations; 2 m (0.5 $L_{s}$) and 2.8 m (0.7 $L_{s}$) stand for the damage at and near the bridge’s midspan, while 0.4 m (0.1 $L_{s}$) represents the damage near the bridge’s support. Common damage like cracks and corrosion (i.e., stiffness and mass reduction) in the material can cause changes in the dynamic properties of the structure, while manually induced damage that increases structural stiffness and mass can also change dynamic characteristics. Mass increases will lead to changes in modal parameters of the bridge (e.g., frequencies), but, more than that, they will change the amplitudes in both time-domain and frequency-domain responses [31]. Thus, one advantage of the ML-based drive-by methods is their ability to detect small-scale damage by tracking the entire frequency or time domain signals. In this study, data from front and rear axle accelerometers are used to build the database. There is a total of 200 (runs) × 4 (cases) × 2 (sensors) = 1600 (signals) in the experimental dataset.

If scaling the proposed approach to real-world cases, for damage detection, the authors would recommend collecting as much “healthy” data as possible, which should include many influencing factors, such as traffic, wind/temperature changes, etc. Damage label data could be hard to obtain in practice. One method is to assume the newly obtained data are damaged, and then use an ML model to classify them and the healthy data [52]. If the accuracy is lower than a threshold (e.g., 60%), they will be marked as healthy; otherwise, they will be marked as damaged. For data processing, a window function that can well handle these noises should be found in the filtering procedure, while the max pooling may still be applicable. These still need further investigation.

## 4. Results and Analysis

This section consists of four subsections. Section 4.1 demonstrates the performance of the algorithm, which is assessed by its improvement in accuracy and efficiency of damage detection with different ML methods. Section 4.2 illustrates the function of the filtering process and discusses the effects of window function parameters. Section 4.3 explains the role of the pooling process and explores the influence of $\overset{-}{N}$ or l. Section 4.4 discusses the results for different sensor locations on the vehicle. At the same time, Section 4.2 and Section 4.3 show how the optimal parameters are chosen by the optimizing operation.

### 4.1. Performance Evaluation of the Proposed Algorithm

**Figure 12:**
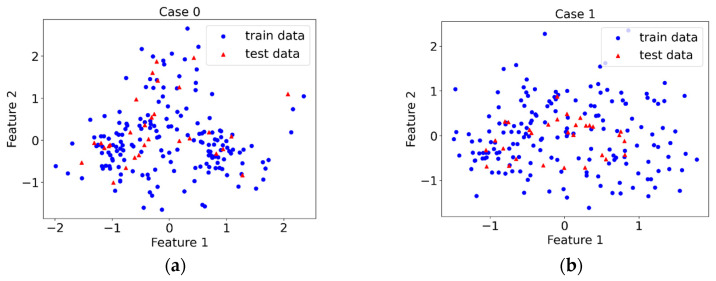

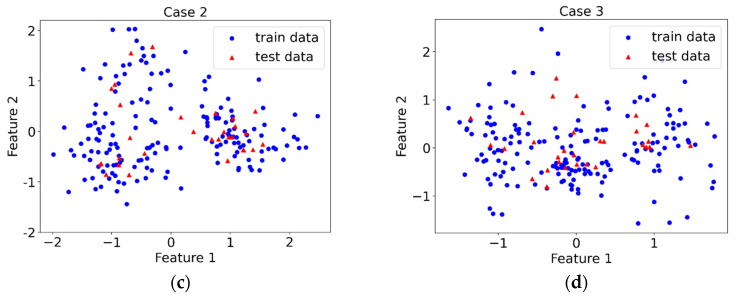
PCA visualization results. (**a**) Case 0, (**b**) Case 1, (**c**) Case 2, (**d**) Case 3.

Principal Component Analysis (PCA) can be used as a visualization tool to present the distribution and variation of data points. PCA converts a given dataset into a new coordinate system by employing an orthogonal linear transformation [53]. The first principal component (feature 1) has the largest variance, followed by the second principal component (feature 2), and so on. The first two features are used to visualize the division of the training set and the test set in each case. It can be found from the visualization results in Figure 12 that there are no two completely overlapping points in each case on the first two features of the training set and the test set. This shows that there is a variation between different drive-by measurements.

The performance of the proposed algorithm is demonstrated by its improvement in accuracy and efficiency of damage detection with different ML models, including Linear-SVM, RBF-SVM, GP, ANN, and RF. Grid Search is used in this study to find the optimal parameters for ML models. The combination that produces the greatest cross-validation score is kept after Grid Search has evaluated all other potential combinations of parameter values in the lists [49]. For different ML models, their main optimal parameters are shown in Table 2. For parameter combinations that have the same scores, the optimum parameters are manually selected from one of them. ANNs may contain complex architectures (e.g., the number of layers and hyperparameters), for which seeking the best or unique solution for a given dataset would be somewhat beyond the scope of this paper. So, only ANNs with simple architectures will be explored.

Table 3 gives the comparison results of ML models with original and processed data as input. For raw data, their data length may be different due to the speed variance. To ensure the fairness of the comparison results, the common time period of each run in the raw time-domain data is used as the input of the ML model, which has about 8000 acceleration points corresponding to 4 s of driving time in this study. Many ML models require their data inputs to be of the same length. For example, the SVM model aims to categorize data points constituted of acceleration amplitudes in a high-dimensional space. If the lengths of the data are different, the spatial dimensions represented by the data points are different, and SVM cannot work at this time. The data processing operations are performed on them. It is deemed that the minimum time period required for the drive-by measurement can be met as long as the chosen time period is longer than one full vibration cycle of the bridge [43]. This can be calculated by $T_{b1}=1/f_{b1}$, or 0.03 s in this study, where $T_{b1}$ is the natural vibration period of the bridge and $f_{b1}$ is the bridge’s first natural frequency. The results with processed data are displayed in bold, and the total time for model training and testing is indicated in parenthesis. All computations in this study were executed in Python 3.8 64-bits on Windows using scikit-learn and SciPy packages [54,55], on a laptop PC with AMD Ryzen 7 CPUs and 8 GB RAM. It should be noted that the results of this subsection and Section 4.2 and Section 4.3 are based on the rear axle sensor, while the results of the front axle sensor will be discussed in Section 4.4. The accuracy improvement in this study refers to the difference in accuracy of the ML model results before and after processing the data. It is clear from the comparison results that both accuracy and efficiency are significantly improved when the processed data are used as input. The proposed method can effectively increase the average accuracy, ranging from 12.2% to 15.0%, in different ML models. Meanwhile, among these ML models, their average runtime decreases by 0.59 to 25.4 s. Their efficiency improvement can be defined as the runtime saved by the processing algorithm (see Equation (5). In the equation, ε represents the efficiency improvement; $t_{O}$ and t represent the total runtime of model training and testing with original and processed data as input, respectively. The proposed method thereby improves the efficiency of ML models by 96.7% (Linear-SVM), 96.7% (RBF-SVM), 59.5% (GP), 91.4% (ANN), and 35.7% (RF), respectively. The runtime of the time-domain signal processing algorithm is 0.19–0.31 s in different cases. By including the signal processing cost in the total computational cost, the overall efficiency can be improved by 64.5% (Linear-SVM), 64.5% (RBF-SVM), 56.3% (GP), 90.7% (ANN), and 30.5% (RF), respectively. Choosing an appropriate and effective data processing method is therefore of great benefit to the data-driven indirect SHM framework. At the same time, it can be found that linear SVM can provide almost the highest accuracy and efficiency among the ML models used. The below results are based on the linear SVM model.

(5) $$\varepsilon =\frac{t_{O}-t}{t_{O}}$$

### 4.2. Discussion on the Filtering Procedure

Section 4.1 validates that the proposed method can effectively improve the accuracy and efficiency of the data-driven indirect SHM framework. In conjunction with the frequency spectrum, this section discusses the function of filtering procedure and the influence of window function parameters.

**Figure 13:**
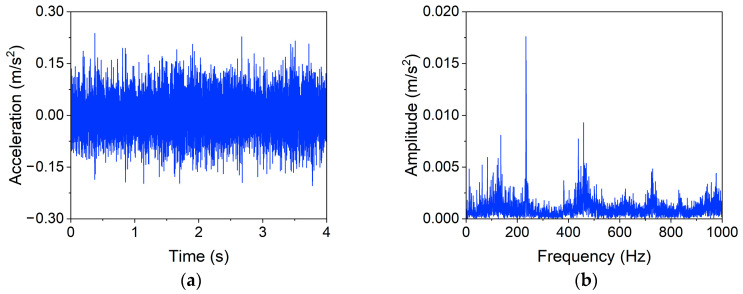
Representative signal before filtering. (**a**) Time domain, (**b**) Frequency domain.

**Figure 14:**
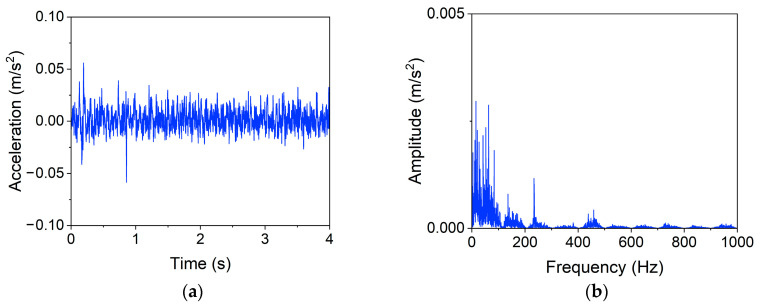
Representative signal after filtering. (**a**) Time domain, (**b**) Frequency domain.

A representative signal with a length of four seconds in the healthy state (case 0) is selected to illustrate the function of the filtering procedure, and similar results can be seen in other cases. Figure 13 shows the time and frequency domain plots of the representative signal before filtering. There are many noisy peaks in the frequency-domain response, especially in the high-frequency region (≥100Hz). It is widely believed that high-frequency signals are usually related to the ambient noise [42]. While, as shown in Figure 14, the peaks of the spectrum are mainly concentrated in the low-frequency region after processing; the filtering operator primarily eliminates the high-frequency noise.

**Figure 15:**
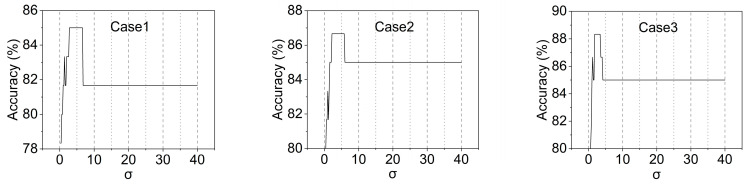
Filtering performance with different σ values.

In this study, the standard Gaussian function is employed as the window function, and its performance is affected by the deviation, σ. Figure 15 shows the accuracies of all three damaged cases corresponding to different σ values, which are results based on the linear SVM model. When the σ value is small, the accuracy sharply rises to a high point as σ increases and remains stable in a certain range (i.e., optimal σ values). Afterwards, the accuracy drops to a lower value and stays constant, but this is still higher than the accuracy of the original data, which shows that data smoothing can improve the accuracy of ML models. For these cases, the optimal σ value lies in the range of 1.8 to 6.6. A common optimal σ value (e.g., σ=3) can thereby be chosen by the optimizing operation as the window function parameter of the filtering procedure.

### 4.3. Discussion on the Pooling Procedure

As can be seen in Section 4.2, the function of the filtering process is to remove noise, which is mainly high-frequency environmental noise in this study. Based on the filtered data, this section explains the function of the pooling procedure, as well as the influence of $\overset{-}{N}$ or l.

**Figure 16:**
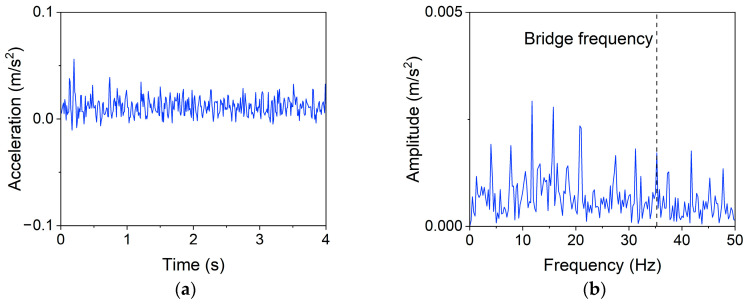
Representative signal after pooling. (**a**) Time domain, (**b**) Frequency domain.

Figure 16 presents the time-domain and frequency-domain responses obtained by pooling the filtered data with a maximum operator that has a window length, l, of 20; the data size after pooling, $\overset{-}{N}$, is 400. It is noticed from the frequency-domain spectrum that the operation removes all frequencies higher than 50 Hz (other frequencies are 0), while the frequency information (e.g., the bridge frequency) lower than 50 Hz is primarily preserved. In other words, it considerably reduces data redundancy while retaining the main characteristics of the data; this is the key to the pooling procedure greatly improving the model efficiency and further boosting the accuracy.

**Figure 17:**
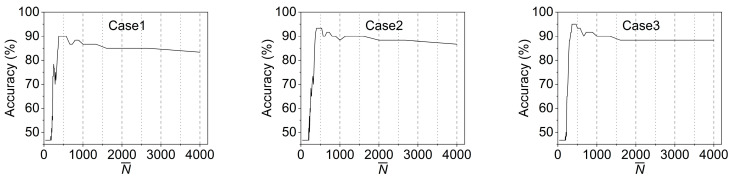
Pooling performance with different data size.

The data size after pooling, $\overset{-}{N}$, and its score on the ML model can be used to intuitively evaluate the performance of the pooling operation. Based on the linear SVM model, the accuracies of three damaged cases with different data sizes are presented in Figure 17. As $\overset{-}{N}$ decreases (or l increases), the accuracy gradually rises, reaches a peak, and then begins to sharply drop. For these cases, peaks are obtained at $\overset{-}{N}$ ranging from 363 to 571 (l ranging from 14 to 22). The optimizing operation will choose one of them as the optimal value (e.g., $\overset{-}{N}=400$). The optimal $\overset{-}{N}$ or l can balance the “complexity” and “diversity” of the data to retain the necessary information while removing noise and redundancy; they can maximize the performance of ML models. It is deemed that a valid drive-by measurement should at least include the fundamental bridge frequency, if only the frequency spectrum is considered. In this study, it can be known from direct measurements that the bridge frequency varies between 33.7 Hz and 35.5 Hz depending on the added mass, which infers that the model score may drop below 60% (close to random guess) with l values between 28 and 30. However, the results show that the accuracy only falls below 60% when the data size is around 222 ($\overset{-}{N}=222$), or l=36 (close to random guess); the pooling operation is no longer meaningful. One explanation is that the time-domain signal is more informative than the frequency-domain signal; for example, damage information may also be contained in local peaks of the time-domain amplitudes. The proposed processing method can largely preserve this temporal information.

**Figure 18:**
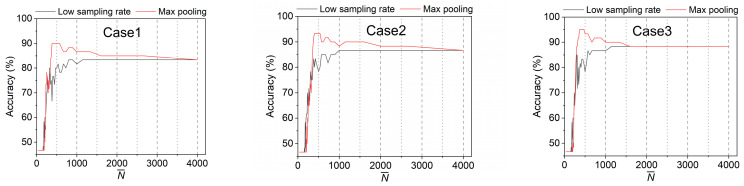
Comparison of low sampling rate and max pooling.

From the comparison of low sampling rate and maximum pooling (see Figure 18), it can be observed that, although the size of the data reduces with the decrease in sampling frequency, the accuracy of damage detection does not decrease within a certain range (e.g., data length ≥ 1500). From the perspective of accuracy, it can be considered that there is data redundancy in the original data under these circumstances. In addition, the max pooling operation is not just simply discarding data like the method of reducing the sampling frequency, but extracting representative features from the data to improve accuracy.

### 4.4. Discussion on the Sensor Location

**Figure 19:**
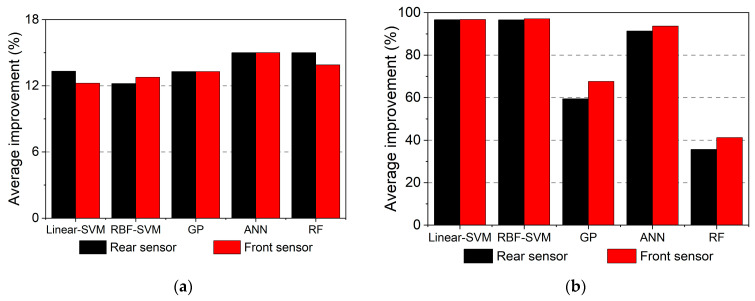
Comparison results for rear and front axles. (**a**) Accuracy, (**b**) Efficiency.

The above results are based on the rear axle sensor, while this section will also compare and discuss the results from the front axle sensor to show the influence of sensor location. Figure 19a shows the comparison results of the average score improvement of different ML models using rear-axle and front-axle data, and the average efficiency improvement can be seen in Figure 19b; the efficiency improvement is calculated using Equation (5). The computing platform of the front axle is the same as that of the rear axle. It is discovered that there are no discernible differences between the two sensors’ accuracy or efficiency improvements. This indicates that the present method’s performance is not significantly affected by the sensor location. In fact, the present data processing algorithm is not strictly limited to any SHM framework or method, but this needs further verification.

## 5. Conclusions

A novel time-domain signal processing algorithm for the raw vehicle accelerations of data-driven drive-by inspection methods is proposed in this paper. It aims to improve the performance of ML-based drive-by methods for detecting bridge damage caused by material deterioration or structural change. The data processing method consists of a filtering process to denoise the data, and a pooling process to equalize the data size and reduce data redundancy. To achieve the best data processing performance, an optimization approach is designed to automatically search for the optimal parameters, tuning the algorithm. The present methodology has been validated via the dataset collected from the laboratorial experiments using a steel beam and a scale truck model. Its performance is demonstrated by its accuracy and efficiency improvement in damage detection with ML models. Based on the results and discussions, the following conclusions can be drawn:

(1) The present algorithm can effectively improve the accuracy and efficiency of different ML models in damage detection. Compared to using raw data, the average accuracy increased by 12.2–15.0%, and the average efficiency increased by 35.7–96.7% for different damaged cases and ML models. This is of great benefit to the data-driven indirect SHM framework.
(2) The filtering procedure primarily eliminates the noise in the data, which is the high-frequency signal associated with ambient noise in this study. There are optimal window function parameters that may achieve the highest accuracy of ML models, but more than that, the results also show that data smoothing can be beneficial for improving accuracy.
(3) The pooling procedure further reduces noise and lessens data redundancy. Appropriate window lengths can balance the “complexity” and “diversity” of the data to retain the necessary information while removing noise and redundancy; they can greatly improve the performance of ML models.
(4) When the proposed method is applied to process data from both the front axle and the rear axle, a similar accuracy or efficiency improvement can be obtained; the algorithm is not significantly affected by the sensor location.

Future work will test the proposed algorithm’s robustness under noise from more varied and complicated sources. The suggested approach may be used with semi-supervised or unsupervised learning techniques to build a new generation of smart bridges for health monitoring that are capable of automatically and accurately detecting damage.

## Figures and Tables

**Table 1:** Case description.

Case No.	Location	Weight	Runs	Case No.	Location	Weight	Runs
0	0	0 (Healthy)	200	2	2 m	20 kg (4%)	200
1	0.4 m	20 kg (4%)	200	3	2.8 m	20 kg (4%)	200

**Table 2:** Major parameters of ML models.

Algorithm	Configuration	Algorithm	Configuration
Linear-SVM	C = 2	ANN	hidden_layer_sizes = (12, 5), alpha = 1, max_iter = 800
RBF-SVM	Gamma = 0.01, C = 2	RF	n_estimators = 900, max_features = 25
GP	Kernel = 100 × RBF (100)		

**Table 3:** Comparison results of accuracy and efficiency.

Case No.	Type	Linear-SVM	RBF-SVM	GP	ANN	RF
Case1	Original	78.3% (0.62 s)	71.7% (0.61 s)	75.0% (6.48 s)	78.3% (28.11 s)	76.7% (4.26 s)
	**Processed**	**90.0% (0.02 s)**	**83.3% (0.02 s)**	**88.3% (2.66 s)**	**91.7% (2.44 s)**	**90.0% (2.72 s)**
Case2	Original	80.0% (0.61 s)	75.0% (0.59 s)	76.7% (6.25 s)	78.3% (27.42 s)	78.3% (4.21 s)
	**Processed**	**93.3% (0.02 s)**	**86.7% (0.02 s)**	**88.3% (2.75 s)**	**93.3% (2.45 s)**	**91.7% (2.79 s)**
Case3	Original	80.0% (0.59 s)	75.0% (0.59 s)	76.7% (6.63 s)	80.0% (27.89 s)	76.7% (4.15 s)
	**Processed**	**95.0% (0.02 s)**	**88.3% (0.02 s)**	**91.7% (2.42 s)**	**96.7% (2.30 s)**	**95.0% (2.61 s)**
Avg. imp.		**13.3% (0.59 s)**	**12.2% (0.58 s)**	**13.3% (3.84 s)**	**15.0% (25.4 s)**	**15.0% (1.5 s)**

## Data Availability

The data used to support the findings of this study are available from the corresponding author upon request.
